# The potential of large rafting objects to spread Lessepsian invaders: the case of a detached buoy

**DOI:** 10.1007/s10530-019-01972-4

**Published:** 2019-03-29

**Authors:** Angelina Ivkić, Jan Steger, Bella S. Galil, Paolo G. Albano

**Affiliations:** 1 Department of Palaeontology, University of Vienna, Althanstrasse 14, 1090 Vienna, Austria; 2 Faculty of Geosciences, Utrecht University, Princetonlaan 8a, 3584 CB Utrecht, The Netherlands; 3 The Steinhardt Museum of Natural History, Israel National Center for Biodiversity Studies, Tel Aviv University, 69978 Tel Aviv, Israel

**Keywords:** Anthropogenic debris, Rafting, Non-indigenous species, Introduction vectors, Fouling, Mediterranean Sea

## Abstract

A diverse and abundant fouling community dominated by Lessepsian non-indigenous species was identified on a 13.5-m-long steel buoy stranded on the Israeli coast but originating from Port Said, at the Mediterranean entrance of the Suez Canal, Egypt. The molluscan community was sampled quantitatively by scraping. Three quarters of the individuals and more than half of the species were non-indigenous. Among the latter, a mytilid bivalve, *Gregariella* cf. *ehrenbergi*, was first recorded in the Mediterranean Sea on the basis of these samples, suggesting that the full consideration of all potential vectors can contribute to non-indigenous species detection. Large floating objects in coastal waters, such as buoys, are particularly suitable for colonization by Lessepsian species because hard substrates, and artificial ones in particular, are highly susceptible to the establishment of non-indigenous species. Moreover, their size and persistence enable the development of abundant and mature fouling communities that can disseminate propagules as eggs and larvae over long distances and for extended periods if detached. This report highlights the potential for large rafting debris as a vector of the spread of non-indigenous biota within the Mediterranean Sea.

## Introduction

Floating marine litter is increasingly becoming an important vector of introduction and spread of non-indigenous species (NIS) (Kiessling et al. 2015). It may double or even triple the dispersal of marine organisms due to its high persistence (Barnes 2002) and open new introduction pathways (Hoeksema et al. 2012, 2015; Holmes et al. 2015; Carlton et al. 2017). The larger and longer-lasting floating objects have a greater potential as vectors for biological invasions because they likely host a more diverse fouling community and may traverse longer distances due to lower degradation and sinkage rates (Thiel and Gutow 2005; Goldstein et al. 2014). A major example is the dispersal of western Pacific species on rafting objects after the tsunami generated by the 2011 East Japan earthquake: 289 Japanese coastal species crossed the northern Pacific Ocean reaching the shores of North America and Hawai’i (Carlton et al. 2017). Some objects were as large as docks or fishing vessels, and able to carry tens of species per object.

In the Mediterranean Sea, the role of marine litter as a primary or secondary vector of species introductions has largely been neglected (Katsanevakis and Crocetta 2014); in the major recent assessments of pathways and vectors in Europe, it has been either lumped with others or not considered (Zenetos et al. 2012; Katsanevakis et al. 2013; Nunes et al. 2014; Galil et al. 2014). To improve our understanding of the role of marine litter as a vector of NIS dispersal, it is necessary to quantify the contribution of litter on their regional spread, determine which litter items are the main carriers, and identify the major donor and recipient areas (Rech et al. 2016).

One of the high-risk donor areas is certainly the Levantine Basin, the easternmost part of the Mediterranean Sea. The opening of the Suez Canal in 1869 triggered the Lessepsian invasion (Por 1978), and the recent Canal enlargement may turn out to have triggered the onset of a new wave of introductions (Galil et al. 2015, 2017). Any floating object in this region may be prone to the colonization by Lessepsian species and consequently may contribute to their transportation to other sectors of the basin.

Thus, the stranding of a 13.5-m-long steel buoy in Israel that was traced to originate from Port Said, Egypt, at the Mediterranean entrance to the Suez Canal, permitted the first examination of the role of large rafting debris in the Lessepsian invasion. During its ca 280-km-long journey, the buoy transported a diverse and abundant invertebrate assemblage dominated by NIS. To our knowledge, this is the first report of large rafting objects as vectors for Lessepsian invaders and our finding clearly demonstrates their potential in facilitating the secondary spread of Lessepsian NIS.

## Materials and methods

In 2014, a 13.5-m-long steel buoy, originally moored at the entrance of the Suez Canal in Port Said, Egypt, got detached and stranded near Shefayim, Israel, from where it was subsequently transported to a dumping site in the close-by Herzliya Marina (Captain M. Solomon and A. Tzindr, pers. comm.). On 28 September 2016, we scraped the fouling community on the buoy from 0.1 m^2^ quadrats approximately every meter along a depth transect from the water line to its lowermost end (at originally 5.5 m depth) and, in addition, a quadrat in the interior of the buoy at ca 5.5 m depth (coded consecutively from Q1, water level, to Q7, in the interior). Q5 lacked fouling organisms and was omitted from consideration; the exterior of the buoy looked abraded, suggesting possible damage during transport with consequent loss of fouling organisms. Due to the long period elapsed between stranding and sampling, soft-bodied organisms were no longer present and thus we focused our research on mollusks, whose shells were abundant, and mostly still retained the dried tissues. The samples were sieved on 0.5 mm and 4 mm mesh and the retained mollusks identified to species. The samples will be deposited in the Natural History Museum Vienna and a voucher collection in the Steinhardt Museum of Natural History, Tel Aviv University.

We computed metrics and drew charts with the statistical programming environment R (R Development Core Team 2008). We created the final graphical output with the program Inkscape.

## Results

We identified 11 indigenous and 10 non-indigenous mollusk species (414 and 1294 individuals, respectively). Bivalves dominated the sampled molluscan community. Among the NIS, we found *Gregariella* cf. *ehrenbergi* (Issel, 1869), a species not previously recorded from the Mediterranean Sea (Steger et al. 2018). On the ground at the Herzliya Marina, next to the buoy, we found specimens of the non-indigenous intertidal gastropod *Cellana rota* (Gmelin, 1791). Fouling gastropods in most cases adhere with their foot and thus fall off once dead. In addition, we found few specimens of the crabs *Eriphia verrucosa* (Forskål, 1775) and the non-indigenous *Sphaerozius nitidus* Stimpson, 1858. The latter was reported from Port Said in a study on brachyuran crabs associated with marine fouling in Mediterranean Egyptian harbors (Ibrahim and Ramadan 2016). Their presence suggests that the buoy arrived in the marina with still living fouling organisms. However, at the time of sampling, the original gastropod assemblage may have been partly lost and therefore be under-represented in our samples, potentially leading to an underestimation of NIS diversity and abundance for this taxonomic group. The buoy was also covered by many individuals of the non-indigenous *Balanus trigonus* (Darwin, 1854) and some specimens of *Perforatus perforatus* (Bruguière, 1789) as well as the non-indigenous *Amphibalanus reticulatus* (Utinomi, 1967).

**Fig. 1:**
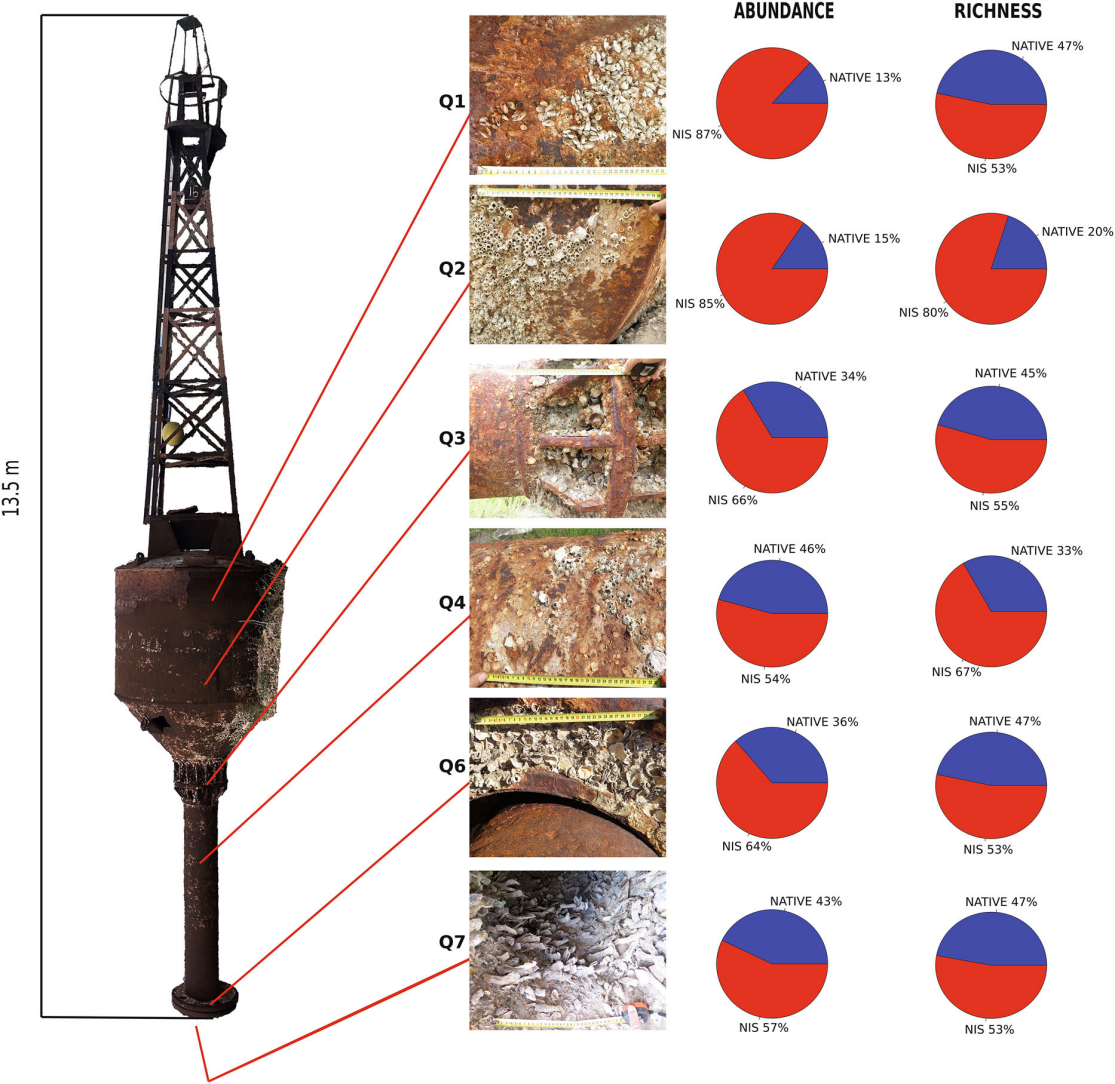
Position of the sampled quadrats on the buoy and share of native versus non-indigenous species (NIS) abundance and richness (in %). Photos were shot with the buoy laying horizontally. The location of Q1 represents the original water line. Q5 was located between Q4 and Q6 but did not contain mollusks and is therefore omitted. Q7 is situated in the interior of the buoy. Abundance and species richness of NIS (in red) were higher than of native species (in blue) at all depths

In all quadrats, NIS were more diverse and abundant than native species, with the highest NIS diversity in Q2 (− 1 m) and the greatest NIS abundance in Q1 (water line) (Fig. 1). The most abundant NIS were *Brachidontes pharaonis* (P. Fischer, 1870) and *Malleus regula* (Forsskål in Niebuhr, 1775) with 746 and 297 individuals, respectively (Table 1). *Modiolus* cf. *barbatus* and *Striarca lactea* (Linnaeus, 1758) were the most abundant native species with 241 and 62 individuals, respectively (Table 1). It is worth mentioning that the identity of the mytilid bivalve *Modiolus* cf. *barbatus* (Linnaeus, 1758) is uncertain: a similar species is known from the Red Sea (Oliver 1992) but the taxonomic knowledge of the genus in the Indo-Pacific province is too poor for a definite identification, especially at the sub-adult stage of most of our specimens. Because of its abundance, however, if this species would prove to be a Red Sea *Modiolus*, most metrics of non-indigenous species diversity and abundance would increase considerably. *Dendostrea* cf. *folium* (Linnaeus, 1758) is also tentatively identified due to the poor taxonomic knowledge of the group (Crocetta et al. 2015), but it is clearly not a native species. In most quadrats, natives were more abundant in the smaller (0.5–4 mm) than in the larger shell size fraction (> 4 mm) (Table 2). The one exception was Q2, a particularly poor sample with only 13 individuals. Overall, species richness did not differ between the size fractions.

**Table 1:** Species abundance in each quadrat (Q5 did not contain any mollusks)

Class	Family	Genus	Status	Q1	Q2	Q3	Q4	Q6	Q7
Gastropoda	*Cerithiopsidae*	*Cerithiopsis tenthrenois* (Melvill, 1896)	NIS	0	0	0	0	0	2
Gastropoda	*Rissoidae*	*Crisilla semistriata* (Montagu, 1808)	Native	0	0	0	0	0	1
Bivalvia	*Arcidae*	*Arca noae* (Linnaeus, 1758)	Native	3	0	0	0	1	0
Bivalvia	*Noetiidae*	*Striarca lactea* (Linnaeus, 1758)	Native	2	**2**	5	1	**29**	23
Bivalvia	*Mytilidae*	*Gregariella* cf. *ehrenbergi* (Issel, 1869)	NIS	12	0	6	2	45	18
Bivalvia	*Mytilidae*	*Musculus subpictus* (Cantraine, 1835)	Native	0	0	0	0	0	2
Bivalvia	*Mytilidae*	*Musculus costulatus* (Risso, 1826)	Native	1	0	0	0	0	0
Bivalvia	*Mytilidae*	*Lithophaga lithophaga* (Linnaeus, 1758)	Native	0	0	1	0	0	4
Bivalvia	*Mytilidae*	*Modiolus* cf. *barbatus* (Linnaeus, 1758)	Native	**83**	0	**49**	**7**	23	**79**
Bivalvia	*Mytilidae*	*Arcuatula senhousia* (Benson, 1842)	NIS	1	0	0	0	0	0
Bivalvia	*Mytilidae*	*Brachidontes pharaonis* (P. Fischer, 1870)	NIS	**708**	**4**	15	1	8	10
Bivalvia	*Mytilidae*	*Septifer cumingii* (Récluz, 1848)	NIS	37	2	4	**3**	20	15
Bivalvia	*Mytilidae*	*Mytilaster* cf. *minimus* (Poli, 1795)	Native	2	0	0	0	1	0
Bivalvia	*Pteriidae*	*Pinctada imbricata radiata* (Leach, 1814)	NIS	3	0	0	0	2	5
Bivalvia	*Ostreidae*	*Ostrea edulis* (Linnaeus, 1758)	Native	27	0	7	1	13	1
Bivalvia	*Ostreidae*	*Dendostrea* cf. *folium* (Linnaeus, 1758)	NIS	5	**4**	28	1	1	7
Bivalvia	*Malleidae*	*Malleus regula* (Forsskål in Niebuhr, 1775)	NIS	58	1	**70**	2	**66**	**100**
Bivalvia	*Chamidae*	*Chama pacifica* (Broderip, 1835)	NIS	3	0	6	1	5	10
Bivalvia	*Myidae*	*Sphenia binghami* (Turton, 1822)	Native	7	0	4	0	16	15
Bivalvia	*Gastrochaenidae*	*Cucurbitula cymbium* (Spengler, 1783)	NIS	0	0	0	0	2	1
Bivalvia	*Gastrochaenidae*	*Rocellaria dubia* (Pennant, 1777)	Native	0	0	0	0	3	1

The most abundant non-indigenous species (NIS) and native species in each quadrat are marked in bold

**Table 2:** Abundance and species richness of non-indigenous species (NIS) and native species in the two size fractions

Quadrat	Mesh size	Abundance		Species richness	
		NIS	Native	NIS	Native
Q1	0.5–4 mm	201 (73%)	75 (27%)	6 (50%)	6 (50%)
	> 4 mm	624 (93%)	49 (7%)	7 (58%)	5 (42%)
Q2	0.5–4 mm	3 (100%)	0 (0%)	1 (100%)	0 (0%)
	> 4 mm	8 (80%)	2 (20%)	4 (80%)	1 (20%)
Q3	0.5–4 mm	29 (37%)	50 (63%)	5 (56%)	4 (44%)
	> 4 mm	98 (87%)	15 (13%)	4 (57%)	3 (43%)
Q4	0.5–4 mm	4 (36%)	7 (64%)	3 (75%)	1 (25%)
	> 4 mm	6 (75%)	2 (25%)	4 (67%)	2 (33%)
Q6	0.5–4 mm	81 (57%)	62 (43%)	7 (54%)	6 (46%)
	> 4 mm	67 (74%)	23 (26%)	24 (86%)	4 (14%)
Q7	0.5–4 mm	75 (38%)	124 (62%)	8 (53%)	7 (47%)
	> 4 mm	93 (98%)	2 (2%)	5 (71%)	2 (29%)

The larger size fraction (> 4 mm) generally shows a greater diversity and abundance of non-indigenous species than the smaller one (0.5–4 mm). Q5 did not contain any mollusks and was therefore omitted

## Discussion

The rich sessile fouling community on the buoy was at an advanced successional stage, as the presence of adult specimens of the bivalves *Brachidontes pharaonis* in Q1 and *Malleus regula* in Q7 suggests (Astudillo et al. 2009). Some specimens of *Pinctada imbricata radiata* are more than 10 cm long and thus more than 6 years old (Narayanan and Michael 1968). Their size, as well as the large size of some specimens of *Chama pacifica* and *Malleus regula*, suggest that this community developed while the buoy was still moored at Port Said.

We also detected a new NIS on the buoy, the mytilid *Gregariella* cf. *ehrenbergi*. This species has a complex taxonomic status and cryptic habitat (Steger et al. 2018) which may cause significant time lags in first detection (Crooks 2005; Albano et al. 2018). Therefore, we cannot determine whether this is truly a recent arrival, but our study demonstrates that the full consideration of all potential vectors can contribute to NIS detection.

Steel buoys are particularly suitable for the colonization by sessile and motile NIS because hard substrates are highly susceptible to their establishment and, once detached from their mooring, are likely to serve as raft for their fouling community (Miller 1968; Kerckhof and Cattrijsse 2001; Wasson et al. 2005; Lim et al. 2009; Nawrot et al. 2015; Simpson et al. 2016). Moreover, artificial substrates facilitate NIS establishment (Bulleri and Airoldi 2005; Glasby et al. 2007; Bulleri and Chapman 2010) because of weaker competitive interactions, as predicted by the biotic resistance hypothesis (Elton 1958), or because of reduced mortality due to predation, as predicted by the enemy release hypothesis (Keane and Crawley 2002) and experimentally tested (Dumont et al. 2011; Rogers et al. 2016). Indeed, 75% of the individuals on the buoy were identified as NIS. The NIS dominance was even more prominent in the larger size fraction (> 4 mm), likely a consequence of the larger average size of the Red Sea species pool (Nawrot et al. 2017).

Marine litter, in general, has rarely been discussed as a vector of primary or secondary NIS introduction in the Mediterranean Sea and, to the best of our knowledge, this is the first report of Lessepsian NIS transported on a detached buoy. The Mediterranean Sea hosts a great number of large floating objects such as navigational buoys, components of aquaculture plants and harbor pontoons, where rich fouling communities can develop (Kerckhof and Cattrijsse 2001; Lim et al. 2009; Rech et al. 2018). If such large objects get detached, they may serve as a vector of NIS spread during rafting because under suitable conditions mature individuals may release eggs and larvae *en route* (Lockwood et al. 2005, 2009; Simberloff 2009). Therefore, we propose that large rafting objects should be fully considered as vectors of biological invasions in the Mediterranean Sea.
